# Exogenous lactobacilli mitigate microbial changes associated with grain fermentation (corn, oats, and wheat) by equine fecal microflora *ex vivo*

**DOI:** 10.1371/journal.pone.0174059

**Published:** 2017-03-30

**Authors:** Brittany E. Harlow, Laurie M. Lawrence, Patricia A. Harris, Glen E. Aiken, Michael D. Flythe

**Affiliations:** 1 Department of Animal and Food Sciences, University of Kentucky, Lexington KY, United States of America; 2 Equine Studies Group, WALTHAM Centre for Pet Nutrition, Melton Mowbray, Leicestershire, United Kingdom; 3 Forage Animal Production Research Unit, Agricultural Research Service, United States Department of Agriculture, Lexington KY, United States of America; Agricultural University of Athens, GREECE

## Abstract

Cereal grains are often included in equine diets. When starch intake exceeds foregut digestion starch will reach the hindgut, impacting microbial ecology. Probiotics (*e*.*g*., lactobacilli) are reported to mitigate GI dysbioses in other species. This study was conducted to determine the effect of exogenous lactobacilli on pH and the growth of amylolytic and lactate-utilizing bacteria. Feces were collected from 3 mature geldings fed grass hay with access to pasture. Fecal microbes were harvested by differential centrifugation, washed, and re-suspended in anaerobic media containing ground corn, wheat, or oats at 1.6% (w/v) starch and one of five treatments: Control (substrate only), *L*. *acidophilus*, *L*. *buchneri*, *L*. *reuteri*, or an equal mixture of all three (10^7^ cells/mL, final concentration). After 24 h of incubation (37°C, 160 rpm), samples were collected for pH and enumerations of total amylolytics, Group D Gram-positive cocci (GPC; Enterococci, *Streptococci*), lactobacilli, and lactate-utilizing bacteria. Enumeration data were log transformed prior to ANOVA (SAS, v. 9.3). Lactobacilli inhibited pH decline in corn and wheat fermentations (P < 0.0001). Specifically, addition of either *L*. *reuteri* or *L*. *acidophilus* was most effective at mitigating pH decline with both corn and wheat fermentation, in which the greatest acidification occurred (P < 0.05). Exogenous lactobacilli decreased amylolytics, while increasing lactate-utilizers in corn and wheat fermentations (P < 0.0001). In oat fermentations, *L*. *acidophilus* and *L*. *reuteri* inhibited pH decline and increased lactate-utilizers while decreasing amylolytics (P < 0.0001). For all substrates, *L*. *reuteri* additions (regardless of viability) had the lowest number of GPC and the highest number of lactobacilli and lactate-utilizers (P < 0.05). There were no additive effects when lactobacilli were mixed. Exogenous lactobacilli decreased the initial (first 8 h) rate of starch catalysis when wheat was the substrate, but did not decrease total (24 h) starch utilization in any case. These results indicate that exogenous lactobacilli can impact the microbial community and pH of cereal grain fermentations by equine fecal microflora *ex vivo*. Additionally, dead (autoclaved) exogenous lactobacilli had similar effects as live lactobacilli on fermentation. This latter result indicates that the mechanism by which lactobacilli impact other amylolytic bacteria is not simple resource competition.

## Introduction

With the use of modern horses for high level performance activities, there has been a concomitant increase in demand to feed horses to maximize their athletic performance. Typically, concentrate is increased and forage is decreased in the diet in order to meet caloric needs. Cereal grains, which are high in starch, are important calorie sources in concentrate feeds used for horses. Previous research in our laboratory demonstrated that grain type can influence microbial changes in equine feces both *ex vivo* [1] and *in vivo* [2]. For example, at equal starch intakes, cracked corn produces more marked changes in the fecal microbial ecosystem than whole cleaned oats, most notably in total amylolytic bacteria (corn: 100,000-fold increase, oats: 10-fold increase). Furthermore, these studies also identified a strong negative correlation between the viable number of lactobacilli and Group D Gram-positive cocci (GPC; *Enterococcus* spp., *Streptococcus bovis/equinus*) and the viable number of lactobacilli and total amylolytic bacteria, indicating a potential competitive relationship between these bacteria in the hindgut. It is noteworthy that these effects were observed both *in vivo* and *ex vivo*, even though the grain substrates in the latter experiments were not subjected to foregut digestion or any simulation thereof.

*Lactobacillus* species are thought to be beneficial to the horse both in regard to their metabolic contribution, but also their important role in competitive exclusion of pathogenic bacteria [3]. For this reason, lactobacilli are often included in probiotic formulations (*e*.*g*., *L*. *acidophilus*). Although, research is limited and existing results are varied on the efficacy of probiotics in horses, some studies have provided evidence that exogenous lactobacilli and other probiotics could have beneficial effects [4, 5].

Certain species of lactobacilli have unique capabilities for ecological competition. For example, *L*. *reuteri*, a member of the equine normal microbiota, can produce antimicrobial molecules with antagonistic activity against *Streptococcus bovis* and *Enterococcus faecalis* [6, 7] Additionally, some species of lactobacilli are unique in that they are not homolactic like most streptococci and lactobacilli. For example, *L*. *buchneri* can produce both acetic and lactic acid during fermentation and also metabolizes lactic acid from the environment into acetic acid under acidic conditions (pH < 5.6, [8, 9]), similar to those found in cases of clinical hindgut acidosis [10]. Therefore, heterofermentative lactobacilli like *L*. *buchneri* could potentially help counteract clinical cases of hindgut acidosis by converting lactic acid into acetic acid, which has a higher pK_a_.

The current study was conducted to determine the effect of exogenous lactobacilli (*L*. *acidophilus*, *L*. *buchneri*, *L*. *reuteri*) on the fermentation of grain by uncultured equine fecal bacteria. The hypothesis was that the addition of lactobacilli could mitigate microbial changes associated with grain fermentation, and these effects would depend on grain type, *Lactobacillus* species and viability. Three grain types (corn, oats and wheat) were included to test for substrate dependence of any effects. It was anticipated that exogenous lactobacilli would impact the pH and the growth of amylolytic bacteria (including GPC and endogenous lactobacilli) and lactate-utilizing bacteria.

## Materials and methods

### Media composition

The cell suspension medium was lightly buffered to allow pH to decrease with fermentation acid production, and contained (per liter): 240 mg KH_2_PO_4_, 240 mg K_2_HPO_4_, 480 mg (NH_4_)_2_SO_4_, 480 mg NaCl, 64 mg CaCl_2_ •2H_2_O, 100 mg MgSO_4_•7H_2_O, 600 mg cysteine hydrochloride; initial pH 6.7; autoclaved to remove O_2_ and cooled under N_2_.

For experiments designed to mimic the equine hindgut environment, a carbonate-buffered cell suspension medium was used [1]. It was prepared as described above, except that the medium was cooled under CO_2_, and 4,000 mg Na_2_CO_3_ was added after cooling.

Growth medium with soluble starch as a substrate (10,000 mg/L) was used for enumeration of total amylolytic bacteria. The growth medium was based on Mantovani and Russell [11] and was heavily buffered to maximize bacterial growth. The medium contained (per liter): 240 mg KH_2_PO_4_, 240 mg K_2_HPO_4_, 480 mg (NH_4_)_2_SO_4_, 480 mg NaCl, 64 mg CaCl_2_ •2H_2_O, 100 mg MgSO_4_•7H_2_O, 600 mg cysteine hydrochloride, 1,000 mg Trypticase, 500 mg yeast extract; initial pH 6.7; autoclaved to remove O_2_ and cooled under O2 –free CO_2_. The buffer (4,000 mg Na_2_CO_3_) was added before dispensing and autoclaving for sterility.

Bile esculin azide agar (Enterococcosel; Becton, Dickinson and Co. (BD), Franklin Lake, NJ, USA) and Rogosa SL agar (BD) were used for aerobic enumeration of Group D Gram-positive cocci (GPC; enterococci and streptococci) and lactobacilli, respectively. Solid media were prepared in Petri plates according to the manufacturer’s directions.

Lactate-utilizing bacteria were enumerated on L-U agar, previously described by Mackie and Heath [12]. L-U agar contained (per liter): 352 mg KH_2_PO_4_, 352 mg K_2_HPO_4_, 704 mg (NH_4_)_2_SO_4_, 704 mg NaCl, 94 mg CaCl_2_•2H_2_O, 147 mg MgSO_4_•7H_2_O, 20,000 mg trypticase, 200 mg yeast extract, 100 mg cysteine hydrochloride, 15,000 mg agar, 220 mmol/L lactic acid, 7.67 μmol/L hemin, 10 mL trace elements solution, 10 mL vitamins solution, and 10 mL VFA solution; initial pH 6.8; autoclaved to remove O_2_ and cooled under O2 –free CO_2_. The buffer (4,000 mg Na_2_CO_3_) was added before Petri plates were prepared in an anaerobic chamber (Coy; Grass Lakes, MI; 95% CO_2_, 5% H_2_).

### Animals and fecal collection

The University of Kentucky Animal Care and Use Committee approved all husbandry and procedures [13]. Mature geldings (n = 7; 5 to 17 y) were selected from the University of Kentucky, Department of Animal and Food Sciences herd at Maine Chance Farm, Lexington KY. The horses selected for the study met the following criteria: no known history of gastrointestinal disease, and no antibiotic or anti-inflammatory chemotherapy for at least 4 months prior to the start of the study. Horses had *ad libitum* access to free choice grass hay with limited access to cool season grass pasture.

When feces were needed for the study, horses were observed in their pasture for defecation. Immediately post-defecation, feces were collected and placed in a plastic bag. Feces were thoroughly mixed by hand. The bag was purged of oxygen with CO_2_ and was then transported in a pre-warmed container (37°C) to the laboratory for processing.

### Fecal cell suspensions

After arrival at the laboratory, fecal bacteria cell suspensions were prepared as previously described [14]. In short, collected feces (450 g) were placed in a blender (continuously sparged with N_2_) and mixed (3 min) with 750 mL of anaerobic cell-suspension medium. The fecal-media mixture was squeezed through 3 layers of cheesecloth to remove plant particles, and subjected to a low-speed centrifugation (341.6 *g*, 5 min) to remove the remaining protists and plant fibers. The collected supernatants then underwent a high-speed centrifugation (25,654.3 *g*, 5 min) to collect prokaryote cells. The remaining supernatants were discarded and the cell pellets were washed by resuspending in anaerobic cell-suspension medium. Prokaryotic cells were then harvested by a second high-speed centrifugation (25,654.3 *g*, 10 min). The supernatants were discarded, and the pellets were re-suspended and pooled into a N_2_-sparged glass bottle (2 L). The optical density of the resulting cell suspension (OD_600_) was adjusted to be ~15. Microscopic analysis revealed prokaryote-sized cells with no obvious plant fiber or protists.

### Effect of starch source and concentration on pH

The grains included finely ground (2 mm screen) minimally processed corn, oats, and wheat. Prior to the start of the study the corn, oats, and wheat to be used were analyzed for chemical composition using commercial wet chemistry methods (Table 1; Dairy One, Ithaca, NY). An initial experiment was conducted to determine the effect of starch concentration on pH of the lightly buffered fecal cell suspensions. Each grain (0 to 2.0% w/v starch, in 0.2% increments, grain weights normalized by starch concentration) was added to a fecal cell suspension that was aliquoted into anaerobic serum bottles. The bottles were incubated in a shaking water bath (37°C, 160 rpm). After 24 h of incubation, samples were collected via tuberculin syringes and the pH was measured immediately with a pH meter. All treatments were performed in duplicate on suspensions made from the feces of three different horses collected on three different days. The starch concentration eliciting maximal effects on pH for all starch sources (1.6% w/v starch) was then selected and used to determine the effects of exogenous lactobacilli addition species and concentration on fecal cell suspension pH.

**Table 1:** Chemical composition: corn, oats, and wheat (As Fed).

	Corn	Oats	Wheat
% DM	89.50	91.90	87.80
DE	3.45	2.68	3.26
% CP	7.50	10.30	10.50
% ADF	3.50	14.40	3.30
% NDF	9.00	28.90	9.80
% Starch	61.80	36.80	58.10
% WSC	2.00	3.20	3.00
% ESC	1.30	2.90	2.50
% Ca	0.01	0.06	0.04
% P	0.22	0.26	0.35

DM: dry matter, DE: estimated digestible energy (Mcal/kg), CP: crude protein, ADF: acid detergent fiber, NDF: neutral detergent fiber, WSC: water soluble carbohydrates, ESC: ethanol soluble carbohydrates, Ca: calcium, P: phosphorus.

### Effect of exogenous lactobacilli addition and concentration on pH

*Lactobacillus acidophilus* (ATCC # 4356), *Lactobacillus buchneri* (ATCC # 4005) and *Lactobacillus reuteri* (ATCC # 23272) type strains were obtained from the American Type Culture Collection (Manassas, VA, USA). Lactobacilli pure cultures were routinely transferred in growth media with glucose as the sole growth substrate (0.4% w/v). When lactobacilli were needed for an experiment, cells from stationary phase (16 h) lactobacilli cultures were harvested by high-speed centrifugation (25,000 *g*, 10 min) in N_2_ –filled Balch tubes. The supernatants were aspirated from the pellet under continuous N_2,_ and re-suspended in anaerobic lightly buffered cell suspension media at 0, 10^2^, 10^4^, 10^6^ or 10^8^ cells/mL.

For each experiment, fecal cell suspensions were dispensed into serum bottles containing ground (2 mm screen) corn, oats or wheat at 1.6% w/v starch concentration (concentration selected from experiment described above). Lactobacilli treatments were then added to the bottles as a 10% v/v addition and the suspensions were incubated as described above for 24 h. Samples were then collected via tuberculin syringes and the pH was measured immediately with a pH meter. All treatments were performed in duplicate on suspensions made from the feces of three different horses collected on three different days.

The exogenous lactobacilli concentration eliciting maximal effects on increasing pH (10^7^ cells/mL, final concentration, for all lactobacilli tested) was then selected and used to determine the effects of *Lactobacillus* spp. and viability (live or dead) on the enumeration of total amylolytic bacteria including, lactobacilli and GPC, and lactate-utilizing bacteria.

### Effect of exogenous *Lactobacillus* spp. and viability on bacterial enumerations

The suspension medium used in the remainder of the experiments was carbonate-buffered cell suspension medium (as described above) to be more representative of the natural environment of the equine hindgut. Lactobacilli additions were prepared as described above, except under a CO_2_-atmosphere, with a final concentration of 10^8^ cells/mL. In addition, paired dead (autoclaved) lactobacilli additions were also prepared (corn only).

Cell suspensions were dispensed into anaerobic serum bottles containing ground (2 mm screen) corn, oats or wheat at 1.6% w/v starch concentration. Lactobacilli treatments were then added to the bottles and the suspensions were incubated as described above for 24 h. Samples were collected via tuberculin syringes after 24 h of incubation for pH, detection and quantification of fermentation end-products and bacterial enumeration. The pH was measured immediately with a pH meter. Supernatants for later fermentation end-product analysis were clarified by centrifugation (21,000 *g*, 2 min), and frozen (-20°C) until analyzed, as described below. Samples for enumerations (1 mL) were serially diluted (10-fold increments) in anaerobic PBS, which was then used to inoculate the selective media. Solid media types were inoculated with 0.2 mL with a sterile spreader. Liquid media types were inoculated with 1 mL with a tuberculin syringe. The experiment was replicated three times with suspensions made from the feces of three different horses.

#### Bacterial enumerations

Total amylolytic bacteria were enumerated in anaerobic liquid medium with soluble starch (as described above). The tubes were incubated (37°C, 3 d). The final dilution exhibiting bacterial growth (viscosity; visual examination) was recorded as the viable number.

Group D Gram-positive cocci (GPC) were enumerated on bile esculin azide agar (Enterococcosel; BD). Lactobacilli were enumerated on Rogosa SL agar (BD). The plates were incubated aerobically (37°C, 3 d). Plates with 30 < × < 300 colonies were considered countable. All colonies on Rogosa agar were counted as lactobacilli. Black colonies on bile esculin azide agar were counted as GPC.

Total lactate-utilizing bacteria were enumerated on L-U agar. The plates were incubated (37°C, 5 d) in an anaerobic chamber (Coy; Grass Lakes, MI; 95% CO_2_, 5% H_2_). Plates with 30 < × < 300 colonies were considered countable. All colonies on L-U agar were counted as lactate-utilizing bacteria.

#### Fermentation end-product analysis by high-performance liquid chromatography (HPLC)

Supernatant samples were thawed and clarified in a micro-centrifuge (21,000 *g*, 2 min). Fermentation end-products (lactate, formate, acetate, propionate, butyrate, EtOH) were quantified using a Summit HPLC (Dionex; Sunnyvale, CA, USA) equipped with an anion exchange column (Aminex HP-87H; Bio-Rad, Hercules, CA, USA) and UV detector. The eluting compounds were separated isocratically with an aqueous sulfuric acid solution (5 mM). The parameters included: injection volume 0.1 mL, flow rate 0.4 mL/min, and column temperature 50°C.

### *Ex vivo* starch disappearance experiments

Finely ground (2 mm screen) corn, oats and wheat were used to determine the effects of exogenous *L*. *reuteri* addition on *ex vivo* starch disappearance. Live and dead (autoclaved) *L*. *reuteri* additions were prepared as described above with a final concentration of 10^8^ cells/mL. Cell suspensions were dispensed into anaerobic Balch tubes containing ground corn, oats or wheat at 1.6% w/v starch concentration. Lactobacilli treatments were then added to the tubes (10% v/v addition; 10^7^ cells/mL, final concentration) and the suspensions were incubated as described above for 24 h. Tubes were destructively sampled and starch was partially hydrolyzed (1:1 addition of cold 1 M acetate buffer, pH 5.0; 200 μL alpha-amylase; incubation at 100°C for 90 min) at 0, 2, 4, 6, 8, and 24 h. Supernatants (50 μL; in duplicate) for later analysis were clarified by centrifugation (3,000 *g*, 10 min), and frozen (-20°C). The experiment was replicated three times with fecal cell suspensions prepared from three different horses.

Starch analysis was performed as described in Sveinbjornsson *et al*. [15]. In short, samples were thawed and starch was fully degraded to glucose (40 μL amyloglucosidase; 200 μL 0.05 M acetate buffer; incubation at 60°C for 60 min). Glucose was then quantified by measuring the increased absorbance of NADPH associated with the reduction of a known quantity of NADP as glucose in the samples is converted to glucose-6-phosphate (abs_1_, 340 nm: 50 μL NADP, 50 μL ATP, 1.45 mL triethanolamine hydrochloride buffer, incubation at room temperature for 5 min; abs_2_, 340 nm: 100 μL hexokinase/glucose– 6- phosphate dehydrogenase; incubation at room temperature for 15 min). The amount of starch in the original sample was then calculated using the final glucose concentration by converting free glucose to starch with a multiplication factor of 0.9.

### Statistical analyses

Prior to statistical analyses, bacterial enumerations were normalized by log transformation. Data (bacterial enumerations, pH, fermentation end-products, % total starch disappearance) were analyzed using the one-way ANOVA procedure of SAS (version 9.3, SAS Inst. Inc., Cary, NY). When a main effect of treatment was detected, means were separated using Fisher’s protected LSD test with statistical significance set at *P* < 0.05. For the lactobacilli concentration experiments, means were separated within *Lactobacillus* species in comparison to substrate only controls. For the remainder of the experiments, means were separated either within starch source or between starch sources depending on the comparisons desired.

Starch disappearance analyses were performed using the MIXED procedure of SAS. The initial rate of starch disappearance was analyzed as a continuous variable (0, 2, 4, 6, 8 h; linear, quadratic and cubic regression coefficients), and treatment as a discrete variable using backward elimination stepwise regression analysis. Models containing only interactions between treatments and regression coefficients were analyzed to determine significant (*P* < 0.05) linear, quadratic, and cubic regression coefficients for each treatment (Little *et al*. 1996). In the presence of a time × treatment interaction, least square means for treatments were compared at each time point using the PDIFF option of SAS.

## Results

**Fig 1:**
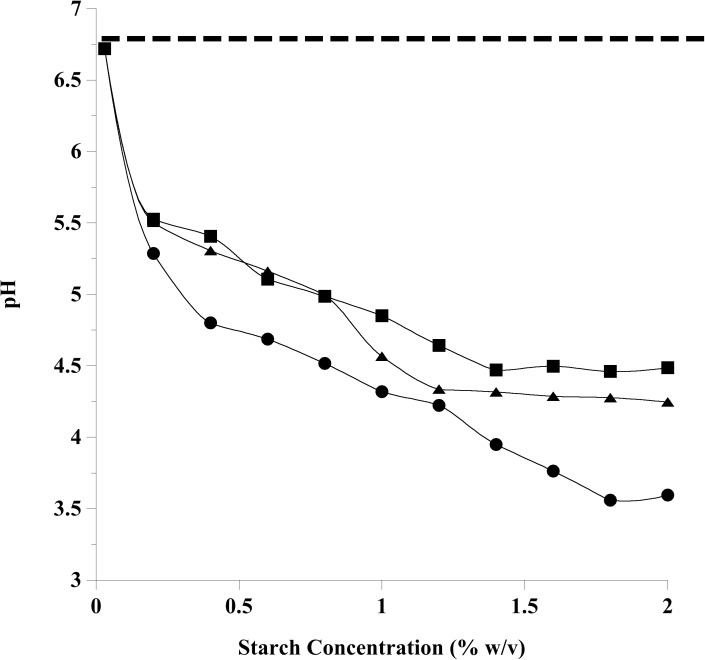
The relationship between pH and starch concentration by grain type after 24 h of fermentation by lightly buffered equine fecal cell suspensions. The suspensions had an initial pH value of 6.8 (dashed line). Starch sources used included finely ground (2 mm screen) corn (circles), oats (squares) and wheat (triangles). Ground grains were included at starch concentrations from 0–2% w/v starch, in 0.2% increments.

When ground corn, oats, or wheat was fermented by equine fecal microflora, the suspension pH declined (Fig 1). The average initial pH values of the cell suspensions were 6.8, and the extent of pH decline over the 24 h incubation was dependent on grain type and starch concentration. The lowest starch concentration eliciting maximal pH effects for all starch sources after 24 h (3.5, 4.4, and 4.2 for corn, oat and wheat incubations, respectively) was 1.6% w/v starch. Based on these results, all subsequent experiments were performed with 1.6% w/v starch.

The effect of exogenous *Lactobacillus* species addition and concentration on pH of fecal cell suspensions fermenting corn, oats or wheat was evaluated after 24 h of incubation. The pH responses were dependent on substrate, *Lactobacillus* species and concentration when 10^2^, 10^4^, and 10^6^ cells/mL exogenous lactobacilli were added to fecal cell suspensions (Table 2). In some cases, these additions led to even greater pH decline than the substrate only control. However, when 10^8^ cells/mL lactobacilli were added exogenously (to achieve 10^7^ cells/mL final conc.) to fecal cell suspensions pH was greater than control (no exogenous lactobacilli), regardless of substrate and *Lactobacillus* species (*P* < 0.05, in all cases). Furthermore, in both corn and wheat incubations (in which the greatest pH decline was observed) the addition of 10^8^ *L*. *reuteri* was most effective at mitigating pH decline (+ 0.2–0.3 pH units; *P* < 0.05; analyses not shown). Therefore, for the remainder of the experiment exogenous lactobacilli were added at a concentration of 10^8^ cells.

**Table 2:** Effect of exogenous lactobacilli addition species and concentration on the pH of equine fecal cell suspensions after 24 h of fermentation of corn, oats or wheat (true means; n = 3).

mmol/L	Cont	10^2^	10^4^	10^6^	10^8^	Sig	SEM
**Corn**							
L.a	3.61^a^	3.61^a^	3.56^b^	3.67^c^	3.75^d^	***	0.0172
L.b	3.61^a^	3.63^a^	3.69^b^	3.63^a^	3.76^c^	***	0.0046
L.r	3.61^a^	3.75^c^	3.65^b^	3.61^a^	3.87^d^	***	0.0058
All	3.61^a^	3.57^b^	3.56^b^	3.60^a^	3.73^c^	***	0.0049
**Oats**							
L.a	4.21^a^	4.26^a^	4.24^a^	4.34^b^	4.40^c^	***	0.0237
L.b	4.21^a^	4.28^b^	4.25^a^	4.24^a^	4.37^c^	***	0.0076
L.r	4.21^a^	4.26^a^	4.28^b^	4.29^b^	4.35^c^	***	0.0041
All	4.21^a^	4.26^a^	4.25^a^	4.27^a^	4.35^b^	**	0.0246
**Wheat**							
L.a	4.10^a^	4.05^b^	4.15^c^	4.19^d^	4.20^d^	**	0.0014
L.b	4.10^a^	4.12^a^	4.11^a^	4.22^b^	4.23^b^	*	0.0126
L.r	4.10^a^	4.20^a^	4.20^a^	4.18^a^	4.32^b^	***	0.0003
All	4.10^a^	4.15^c^	4.04^b^	4.13^a^	4.20^d^	**	0.0067

Cont: Control (grain only); L.a: *Lactobacillus acidophilus*; L.b: *Lactobacillus buchneri*; L.r: *Lactobacillus reuteri*; All: *L*. *acidophilus*, *L*. *buchneri*, and *L*. *reuteri* (at equal concentrations).

Values with different markers (^a,b,c,d^) are statistically different (***P < 0.0001; **P < 0.001; *P < 0.05).

**Fig 2:**
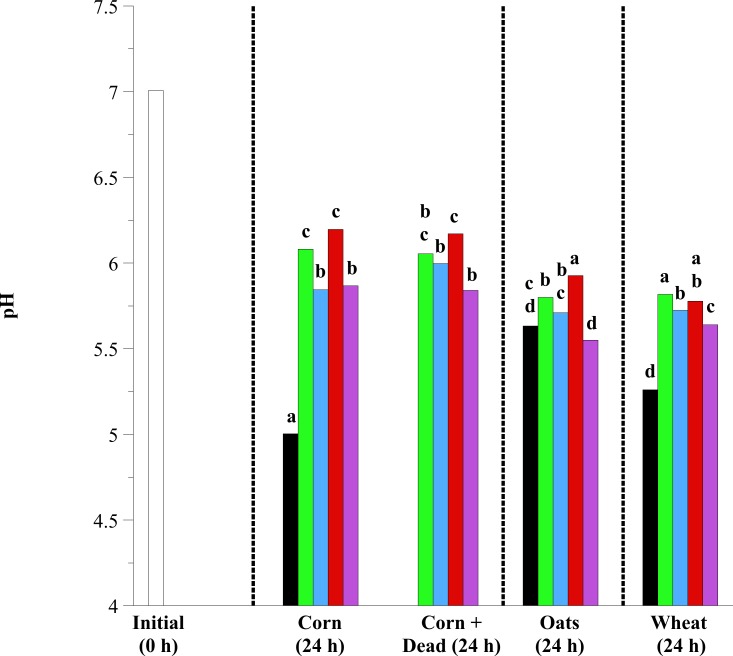
The effect of exogenous lactobacilli addition on the pH of equine fecal cell suspensions. Grain types included minimally processed, finely ground (2 mm screen) corn, oats and wheat at 1.6% w/v starch concentration. Treatments included initial (open bars; 0 h), control (black bars; substrate only), and the addition of *L*. *acidophilus* (green bars), *L*. *buchneri* (blue bars), *L*. *reuteri* (red bars) and Mixed (purple bars; all 3 at equal concentrations) at 10^7^ final concentration live or dead (autoclaved; corn only). Samples were taken after 24 h of incubation (37°C) for pH. Hatched lines separate individual statistical comparisons. Means lacking a common letter are different between treatments within substrate (*P* < 0.05); Corn: treatment, *P* < 0.0001; Oats: treatment, *P* = 0.0012; Wheat: treatment, *P* = 0.0012; Pooled SEM Corn: treatment = 0.0674; Oats: treatment = 0.0187; Wheat: treatment = 0.0349.

Based on the aforementioned observations, an experiment was conducted to determine the effect of exogenous *Lactobacillus* species addition and viability (live vs. dead) on pH, fermentation end-product concentrations and the growth of amylolytic bacteria (including lactobacilli and GPC) and lactate-utilizing bacteria. Similar to previous experiments, the addition of 10^8^ exogenous lactobacilli, regardless of species and viability (10^8^ before heat kill, 0 after heat kill), inhibited the pH decrease of fecal cell suspensions fermenting corn (*P* < 0.0001), oats (*P* = 0.0012) or wheat (*P* = 0.0012; except Mixed treatment in oat fermentations; Fig 2). *L*. *acidophilus* and *L*. *reuteri* were most effective at mitigating pH decline with starch source fermentation (*P* < 0.05). For example, in corn fermentations the addition of exogenous *L*. *reuteri* increased suspension final pH by 1.2 units (pH 5.0 in control; pH 6.2 with *L*. *reuteri* addition).

The addition of 10^8^ lactobacilli (10^7^, final concentration), regardless of species and viability, decreased lactate concentrations in fecal cell suspensions fermenting corn (*P* < 0.0001), oats (*P* < 0.0001) or wheat (*P* < 0.0001; except *L*. *buchneri* and Mixed treatments in oat fermentations; Table 3). For all grain types, *L*. *acidophilus* and *L*. *reuteri* additions were most effective at reducing lactate concentrations, regardless of viability (*P* < 0.05). For example, in corn fermentations live or dead *L*. *acidophilus* and *L*. *reuteri* additions reduced lactate concentrations by as much as 85%. Substrate- and inoculum-dependent results were observed for the additional fermentation end-products detected (formate, acetate, propionate, butyrate, EtOH).

**Table 3:** Effect of exogenous lactobacilli addition (10^8^; live or dead) on fermentation end-product production by equine fecal cell suspensions after 24 h of fermentation of corn, oats or wheat (true means; n = 3).

mmol/L	Cont	L.a	L.b	L.r	All	L.a (dead)	L.b (dead)	L.r (dead)	Mixed (dead)	Sig
**Corn**										
Lactate	11.4^a^	1.7^d^	6.3^b^	1.0^d^	6.0^b^	1.2^d^	5.0^bc^	1.2^d^	4.6^c^	***
Formate	4.9^a^	0.0^b^	0.3^b^	0.0^b^	0.8^b^	0.0^b^	3.9^a^	1.1^b^	1.0^b^	***
Acetate	13.3^cd^	15.3^bc^	15.0^bc^	19.7^ab^	9.7^d^	18.3^b^	15.0^bc^	24.3^a^	18.0^bc^	**
Propionate	14.0^de^	21.0^bc^	16.7^cd^	24.7^ab^	11.2^e^	22.3^b^	19.7^bc^	28.3^a^	21.7^bc^	***
Butyrate	0.8^d^	3.6^bc^	2.6^cd^	3.9^bc^	2.2^cd^	5.6^b^	2.9^cd^	9.2^a^	4.2^bc^	***
EtOH	1.8^a^	0.0^c^	1.5^ab^	0.8^bc^	0.3^c^	0.0^c^	0.8^bc^	0.0^c^	0.7^c^	*
**Oats**										
Lactate	7.3^a^	1.2^c^	8.7^b^	1.9^d^	9.1^b^	-	-	-	-	***
Formate	0.0^b^	1.0^b^	4.2^a^	0.3^b^	4.6^a^	-	-	-	-	*
Acetate	18.0^a^	13.3^bc^	16.5^a^	13.7^b^	11.3^c^	-	-	-	-	*
Propionate	19.0^a^	19.0^a^	13.0^b^	18.7^a^	11.0^b^	-	-	-	-	*
Butyrate	3.4^a^	2.2^ab^	0.8^b^	3.5^a^	0.4^b^	-	-	-	-	*
EtOH	1.5	0.8	1.3	0.4	1.9	-	-	-	-	ns
**Wheat**										
Lactate	9.2^c^	2.5^e^	6.2^b^	1.2^d^	6.8^a^	-	-	-	-	***
Formate	2.5	1.7	1.1	0.0	1.1	-	-	-	-	ns
Acetate	14.0^a^	10.0^b^	10.3^b^	16.7^a^	8.7^b^	-	-	-	-	*
Propionate	16.0^ab^	10.5^b^	8.0^b^	20.7^a^	9.3^b^	-	-	-	-	*
Butyrate	1.9^b^	2.7^b^	1.5^b^	5.1^a^	1.3^b^	-	-	-	-	*
EtOH	1.9^a^	0.0^b^	1.1^a^	0.0^b^	1.0^a^	-	-	-	-	*

Cont: Control (grain only); L.a: *Lactobacillus acidophilus*; L.b: *Lactobacillus buchneri*; L.r: *Lactobacillus reuteri*; All: *L*. *acidophilus*, *L*. *buchneri*, and *L*. *reuteri* (at equal concentrations).

Values with different markers (^a,b,c,d,e^) are statistically different (***P < 0.0001; **P < 0.001; *P < 0.05).

Quantities < 1.0 mmol/L were considered below the limit of quantification (trace) and included as 1 mmol/L) in the statistical analysis.

(Corn) SEM: lactate = 0.601; formate = 0.719; acetate = 8.009; propionate = 8.357; butyrate = 1.763; EtOH = 0.223

(Oats) SEM: lactate = 0.030; formate = 0.759; acetate = 1.068; propionate = 3.274; butyrate = 0.535; EtOH = 0.495

(Wheat) SEM: lactate = 0.283; formate = 3.487; acetate = 5.73; propionate = 4.898; butyrate = 1.593; EtOH = 0.279

**Fig 3:**
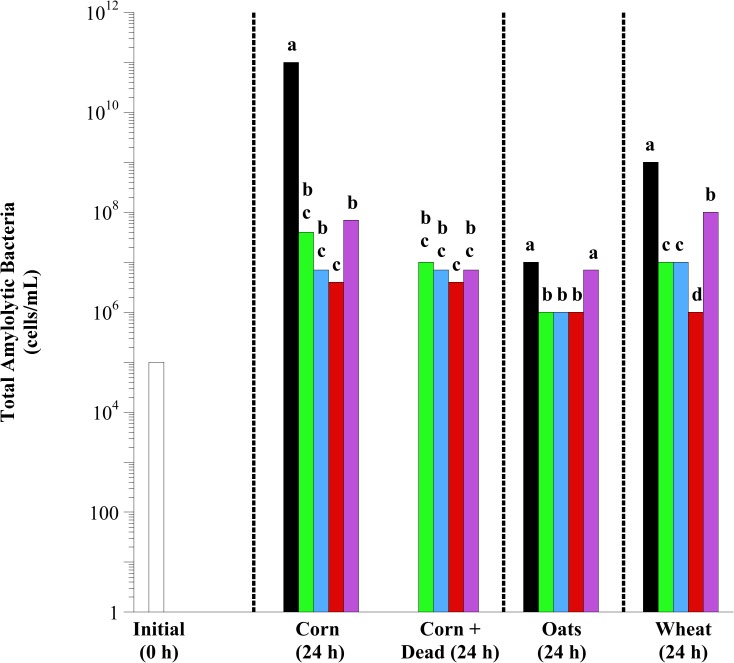
The effect of exogenous lactobacilli addition on the viable number of total amylolytic bacteria in equine fecal cell suspensions. Grain types included minimally processed, finely ground (2 mm screen) corn, oats and wheat at 1.6% w/v starch concentration. Treatments included initial (open bars; 0 h), control (black bars; substrate only), and the addition of *L*. *acidophilus* (green bars), *L*. *buchneri* (blue bars), *L*. *reuteri* (red bars) and Mixed (purple bars; all 3 at equal concentrations) at 10^7^ final concentration live or dead (autoclaved; corn only). Samples were taken after 24 h of incubation (37°C) for bacterial enumeration. The enumerations were performed in anaerobic liquid media with soluble starch as the growth substrate. The tubes were incubated (37°C, 3 d), and the final dilution exhibiting bacterial growth (visual examination) was recorded as the viable number. Hatched lines separate individual statistical comparisons. Means lacking a common letter are different between treatments within substrate (*P* < 0.05); Corn: treatment, *P* < 0.0001; Oats: treatment, *P* = 0.0033; Wheat: treatment, *P* < 0.0001; Pooled SEM Corn: treatment = 0.4513; Oats: treatment = 0.2582; Wheat: treatment = 0.2582 (log_10_ transformed).

Initially, 10^5^ amylolytic bacteria were observed in fecal cell suspensions (Fig 3). After 24 h of incubation, exogenous lactobacilli addition, regardless of species and viability, decreased the viable number of total amylolytic bacteria observed with corn (*P* < 0.0001), oat (*P* = 0.0033) and wheat (*P* < 0.0001) fermentation (except Mixed with oats). In both corn and wheat fermentations, *L*. *reuteri* addition was most effective at mitigating total amylolytic bacteria proliferation (10,000- and 100- fold in corn and wheat incubations, respectively; *P* < 0.05). Furthermore, in corn fermentations, lactobacilli additions had equal efficacy when added live or dead (*P* < 0.05). The effect of lactobacilli viability was only tested in corn fermentations and not in oat or wheat fermentations.

**Fig 4:**
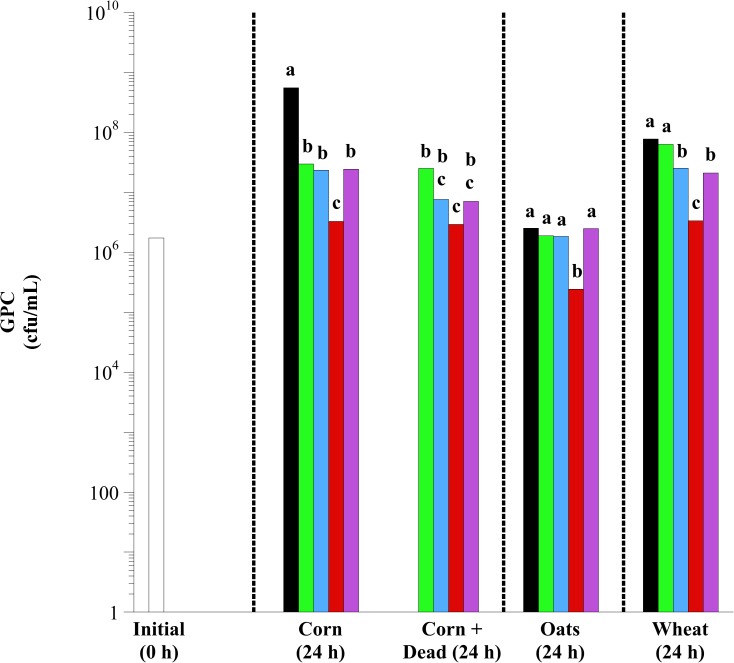
The effect of exogenous lactobacilli addition on the viable number of Group D Gram-positive cocci (GPC) in equine fecal cell suspensions. Grain types included minimally processed, finely ground (2 mm screen) corn, oats and wheat at 1.6% w/v starch concentration. Treatments included initial (open bars; 0 h), control (black bars; substrate only), and the addition of *L*. *acidophilus* (green bars), *L*. *buchneri* (blue bars), *L*. *reuteri* (red bars) and Mixed (purple bars; all 3 at equal concentrations) at 10^7^ final concentration live or dead (autoclaved; corn only). Samples were taken after 24 h of incubation (37°C) for bacterial enumeration. GPC were enumerated on bile esculin azide agar (BD). The plates were incubated aerobically (37°C, 3 d). Plates with 30 < x < 300 colonies were counted. Black colonies on bile esculin azide agar were counted as GPC. Hatched lines separate individual statistical comparisons. Means lacking a common letter are different between treatments within substrate (*P* < 0.05); Corn: treatment, *P* < 0.0001; Oats: treatment, *P* < 0.0001; Wheat: treatment, *P* < 0.0001; Pooled SEM Corn: treatment = 0.1190; Oats: treatment = 0.0859; Wheat: treatment = 0.0091 (log_10_ transformed).

**Fig 5:**
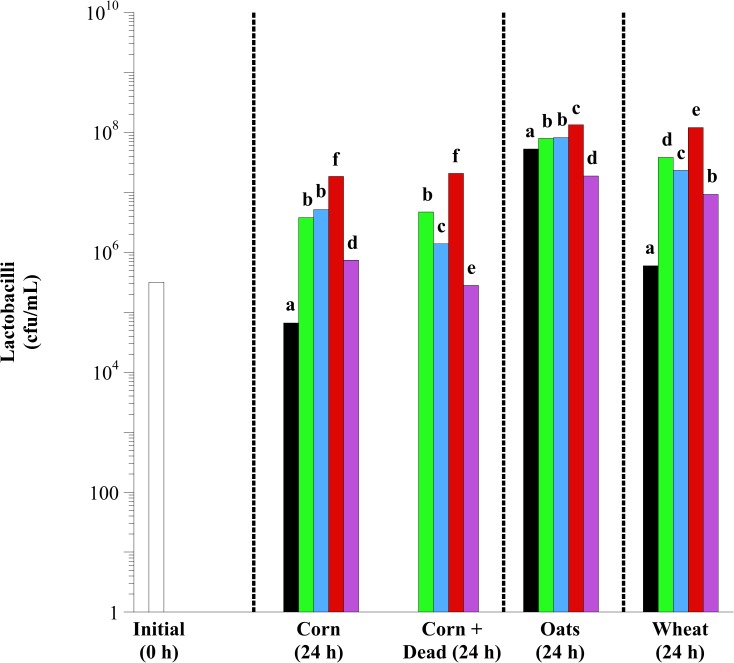
The effect of exogenous lactobacilli addition on the viable number of lactobacilli in equine fecal cell suspensions. Grain types included minimally processed, finely ground (2 mm screen) corn, oats and wheat at 1.6% w/v starch concentration. Treatments included initial (open bars; 0 h), control (black bars; substrate only), and the addition of *L*. *acidophilus* (green bars), *L*. *buchneri* (blue bars), *L*. *reuteri* (red bars) and Mixed (purple bars; all 3 at equal concentrations) at 10^7^ final concentration live or dead (autoclaved; corn only). Samples were taken after 24 h of incubation (37°C) for bacterial enumeration. The enumerations were performed on Rogosa SL agar (BD). The plates were incubated aerobically (37°C, 3 d). Plates with 30 < x < 300 colonies were counted. All colonies on Rogosa SL agar were counted as lactobacilli. Hatched lines separate individual statistical comparisons. Means lacking a common letter are different between treatments within substrate (*P* < 0.05); Corn: treatment, *P* < 0.0001; Oats: treatment, *P* < 0.0001; Wheat: treatment, *P* < 0.0001; Pooled SEM Corn: treatment = 0.1042; Oats: treatment = 0.0548; Wheat: treatment = 0.0624 (log_10_ transformed).

In initial equine fecal cell suspensions, 1.7 × 10^6^ to 1.8 × 10^6^ and 3.1 × 10^5^ to 3.3 × 10^5^ GPC (Fig 4) and lactobacilli (Fig 5) were observed, respectively. There were effects of lactobacilli treatment on GPC and lactobacilli enumerations in corn, oat and wheat incubations (*P* < 0.0001, in all cases). Exogenous lactobacilli addition decreased the growth of GPC and increased the growth of lactobacilli. However, these effects were both species- and substrate-dependent. For example, *L*. *acidophilus* addition was only effective at decreasing GPC growth in corn fermentations. Additionally, there was no additive effect of combining the *Lactobacillus* species together (Mixed). In fact, the Mixed treatment was consistently the least effective at increasing the viable number of lactobacilli with any grain type. In oat fermentations specifically, Mixed decreased the viable number of lactobacilli in comparison to control (substrate only; *P* < 0.05). Exogenous addition of *L*. *reuteri* was most effective at decreasing the viable number of GPC and increasing the viable number of lactobacilli (*P* < 0.05). In corn fermentations, *L*. *reuteri* decreased enumerable GPC and increased enumerable lactobacilli by > 100-fold, regardless of viability (*P* < 0.05).

**Fig 6:**
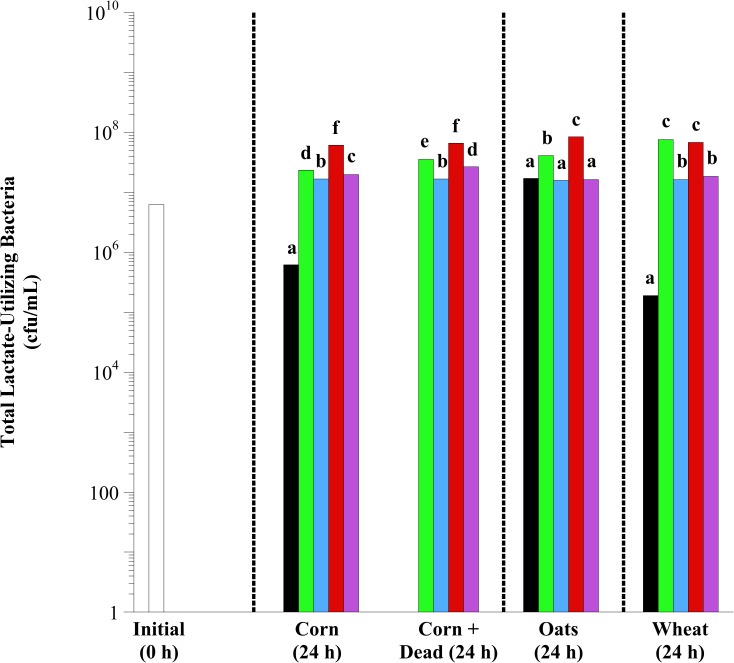
The effect of exogenous lactobacilli addition on the viable number of total lactate-utilizing bacteria in equine fecal cell suspensions. Grain types included minimally processed, finely ground (2 mm screen) corn, oats and wheat at 1.6% w/v starch concentration. Treatments included initial (open bars; 0 h), control (black bars; substrate only), and the addition of *L*. *acidophilus* (green bars), *L*. *buchneri* (blue bars), *L*. *reuteri* (red bars) and Mixed (purple bars; all 3 at equal concentrations) at 10^7^ final concentration live or dead (autoclaved; corn only). Samples were taken after 24 h of incubation (37°C) for bacterial enumeration. Total lactate-utilizing bacteria were enumerated on L-U agar. The plates were incubated anaerobically (37°C, 5 d). Plates with 30 < x < 300 colonies were counted. All colonies on L-U agar were counted as lactate-utilizing bacteria. Hatched lines separate individual statistical comparisons. Means lacking a common letter are different between treatments within substrate (*P* < 0.05); Corn: treatment, *P* < 0.0001; Oats: treatment, *P* < 0.0001; Wheat: treatment, *P* < 0.0001; Pooled SEM Corn: treatment = 0.0390; Oats: treatment = 0.0437; Wheat: treatment = 0.0329 (log_10_ transformed).

In initial fecal cell suspensions, 6.15 × 10^6^ to 6.34 × 10^6^ total lactate-utilizing bacteria were observed (Fig 6). After 24 h of incubation, lactobacilli addition, regardless of species and viability, increased the viable number of total lactate-utilizing bacteria observed with corn (*P* < 0.0001), oat (*P* < 0.0001) and wheat (*P* < 0.0001) fermentation (except *L*. *buchneri* and Mixed with oats). In both corn and oat fermentations, *L*. *reuteri* addition was most effective at increasing the viable number of total lactate-utilizing bacteria (*P* < 0.05). In wheat fermentations, *L*. *acidophilus* and *L*. *reuteri* additions were equally effective. Based on the aforementioned results, exogenous *L*. *reuteri* (regardless of viability) was most consistently effective at mitigating changes associated with starch fermentation. However, the mechanism of action of how this mitigation occurs is unclear. Therefore, the subsequent experiment was conducted to determine how *L*. *reuteri* addition (live and dead) impacts starch fermentation by equine fecal microflora.

**Fig 7:**
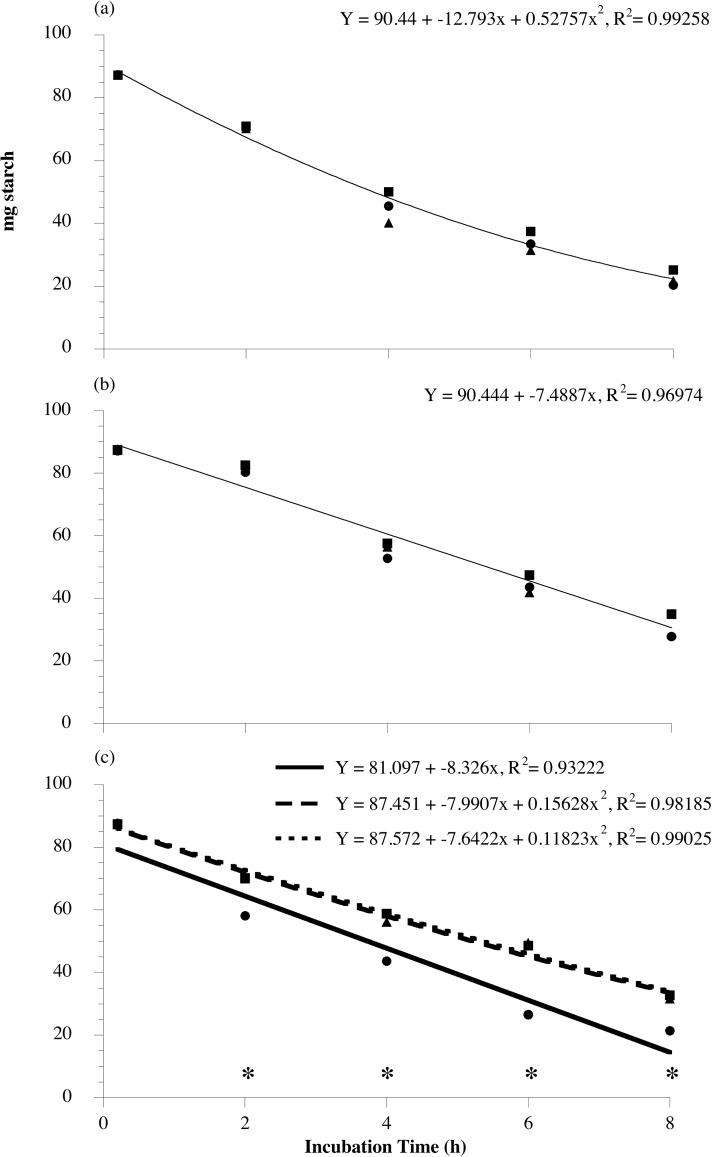
The effect of exogenous *Lactobacillus reuteri* addition on the initial rate of starch disappearance by equine fecal cell suspensions. Grain types included minimally processed, finely ground (2 mm screen) corn (a), oats (b), and wheat (c) at 1.6% w/v starch concentration. The treatments included substrate only (circles, solid line), substrate + 10^8^ *L*. *reuteri* live (squares, hatched line; 10^7^ final concentration) or substrate + 10^8^ *L*. *reuteri* dead (autoclaved; triangles, dotted line). Samples for starch analysis were taken at 0, 2, 4, 6, and 8 h of incubation. Asterisks indicate a significant difference between *L*. *reuteri* (live or dead) treated suspensions and the substrate only control within a time point (*P* < 0.0001); Corn: *P* = 0.0796; Oats: *P* = 0.4568; Wheat: *P* < 0.0001; SE Corn = 2.6373; Oats = 0.6326; Wheat = 1.9951.

Starch disappearance, from 0–8 h of incubation, showed decreases in substrate only controls over time. Starch disappearance in corn incubations was faster than in oat incubations, with wheat being intermediate (P < 0.0001). No effect of *L*. *reuteri* addition (live or dead) on the initial rate of starch disappearance was observed in corn (overall quadratic model for all treatments; 90.44 + -12.79x + 0.52757x^2^; Fig 7A) or oat incubations (overall linear model for all treatments; 90.444 + -7.4887x; P > 0.05, in all cases; Fig 7B). However, wheat fermentations with added live (quadratic model; 87.451 + -7.9907x + 0.15628x^2^) or dead (quadratic model; 87.527 + -7.6422x + 0.11823x^2^) *L*. *reuteri* had a slower rate of starch disappearance than the substrate only control (linear model: 81.097 + -8.326x) (P < 0.0001; Fig 7C). Furthermore, differences in the rate of starch disappearance resulted in the *L*. *reuteri* treated suspensions having higher concentrations of starch at 2, 4, 6, and 8 h of incubation in comparison to control (P < 0.0001, in all cases).

**Fig 8:**
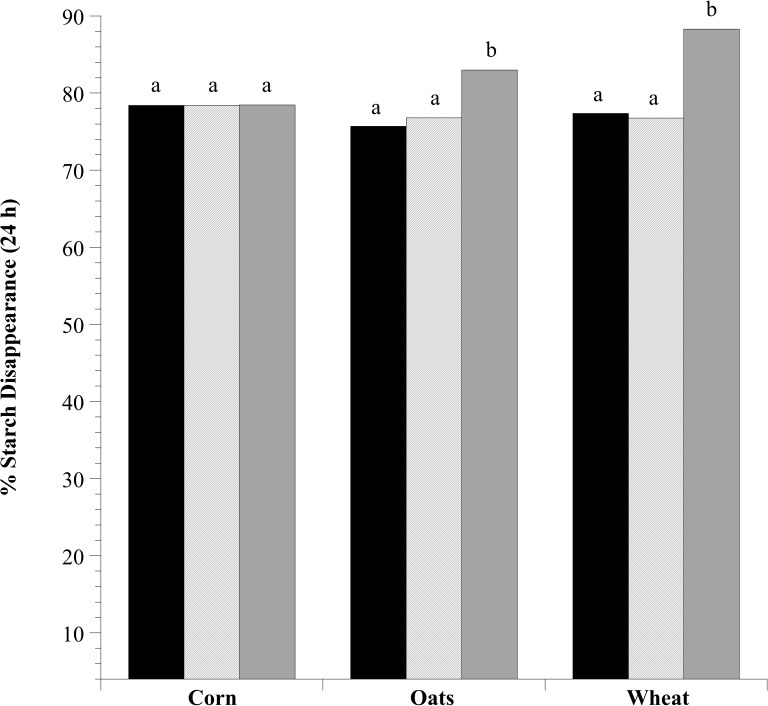
The effect of exogenous *Lactobacillus reuteri* addition on the % total starch disappearance by equine fecal cell suspensions. Grain types included minimally processed, finely ground (2 mm screen) corn, oats and wheat at 1.6% w/v starch concentration. The treatments included substrate only (black), substrate + 10^8^ *L*. *reuteri* live (hatched; 10^7^ final concentration) or substrate + 10^8^ *L*. *reuteri* dead (autoclaved; grey). Samples for starch analysis were taken at 0 and 24 h of incubation. Means lacking a common letter are different between treatments within substrate (*P* < 0.05); Corn: treatment, *P* = 0.8898; Oats: treatment, *P* < 0.0001; Wheat: treatment, *P* < 0.0001; Pooled SEM Corn: treatment = 0.0390; Oats: treatment = 0.0437; Wheat: treatment = 0.0329.

The percentage of total starch disappearance after 24 h of incubation was similar in corn, oat and wheat incubations (>75%, in all cases; *P* > 0.05). However, fermentations containing added *L*. *reuteri* either had similar or higher total starch disappearance in comparison to substrate only controls (Corn: *P* = 0.8898; Oats: *P* < 0.0001; Wheat: *P* < 0.0001; Fig 8). In all cases, fermentations with added live *L*. *reuteri* had similar total starch disappearance to substrate only controls (*P* > 0.05). In contrast, additions of dead *L*. *reuteri* caused an increase in percent total starch disappearance in oat (~83%) and wheat (~88%) incubations (*P* < 0.05).

## Discussion

It is counter-intuitive to propose inhibiting starch fermentation by adding a starch-fermenting organism, like a lactobacillus. However, previous studies in our laboratory have identified a strong negative relationship between the number of lactobacilli and the total number of amylolytic bacteria with grain fermentation, indicating competitive relationships among these bacteria (Harlow *et al*. 2015; Harlow *et al*. 2016). Based on the aforementioned observations, the objective of the current study was to determine if exogenous lactobacilli additions could mitigate both pH and microbial changes associated with corn, oat and wheat fermentation *ex vivo*.

The results from the grain only fermentations (controls) in the current study are consistent with previous reports; i.e., different grains promoted the growth of bacterial guilds to different extents [1, 2]. Notably, corn fermentations had the lowest pH and promoted the growth of total amylolytic bacteria and GPC while decreasing lactobacilli and lactate-utilizing bacteria. Wheat produced similar results to corn, except both GPC and lactobacilli increased in wheat fermentations. In contrast, fermentation with oats had the highest pH, and favored the growth of lactobacilli and lactate-utilizing bacteria while inhibiting GPC. Corn also had the fastest rate of starch disappearance, oats the slowest, and wheat fermentations were intermediate. Decreasing the rate of starch disappearance could allow more time for adaptation of the hindgut microflora and consequent increased stability. This latter idea is consistent with the observation that total percent starch disappearance was similar for all starch sources. Therefore, consequent substrate availability from starch fermentation for energetic end-product conversion to meet the horse’s energy requirements may not be affected by starch source. However, future research is needed to evaluate this phenomenon *in vivo*.

The addition of exogenous lactobacilli mitigated pH and microbial changes associated with corn, oat and wheat fermentation. The effects depended on concentration, species and substrate, but not on the viability of the lactobacilli. Autoclaved lactobacilli were just as inhibitory to pH decline as live. Amelioration of pH decline was lactobacilli addition concentration dependent. In fact, in some cases additions < 10^8^ cells/mL led to greater pH decline, while 10^8^ cells/mL additions increased pH relative to substrate only controls. It is important to consider that lactobacilli are amylolytic bacteria that produce lactic acid, so it is reasonable that they would decrease pH. Considered together, 1) the dose-response relationship and 2) the inconsequence of viability, indicate that the mechanism by which pH decline was inhibited could be pre-formed antimicrobial compounds. Therefore, exogenous lactobacilli at lower concentrations could have grown *in situ*, contributing greater lactic acid production and consequent pH decline. In contrast, the addition of lactobacilli at higher concentrations could have an antimicrobial effect on more efficient amylolytic bacteria, decreasing lactate production and pH decline. Previous studies demonstrated that 10^9^ to 10^10^ bacteria were required to observe any beneficial health effects of probiotics, with lower levels showing little to no effects [16].

*Lactobacillus reuteri* addition was the most consistently effective species in the current study. In corn fermentations, *L*. *reuteri* addition increased pH (+ 0.3 units), acetate (~ 32%), propionate (~ 43%), butytrate (~ 80%), lactobacilli (>100-fold), and lactate-utilizing bacteria (>100-fold) while decreasing lactate (~ 90%), GPC (>100-fold) and total amylolytic bacteria (>10,000-fold). Additionally, the effects observed with *L*. *reuteri* addition were the same regardless of viability. Interestingly, addition of *L*. *reuteri* (regardless of viability) decreased the rate of starch disappearance with wheat fermentation but not with corn or oat fermentation. Despite these differences, at 24 h fermentations with added live or dead *L*. *reuteri* had similar or greater total starch disappearance as the substrate only controls, respectively.

*Lactobacillus reuteri* is highly abundant in the normal equine hindgut microflora [7], and has been shown to survive passage through the stomach and upper small intestine and transiently colonize the gastrointestinal tract in humans, making it a prime probiotic candidate for use in horses [17]. Furthermore, this bacterium has been used for > 20 years as a probiotic and/or starter culture in food and health care products [18]. *Lactobacillus reuteri* has the ability to synthesize 3-hydroxypropionalehyde (reuterin) as a by-product of glycerol fermentation. Reuterin is a potent antimicrobial agent active against a broad spectrum of microorganisms including *Streptococcus* spp. and *Enterococcus* spp. [19]. Furthermore, reuterin is water-soluble and is highly effective at low pH values like those encountered in the acidotic hindgut [20, 18].

Another interesting observation made in the current study was combining the lactobacilli species did not have additive effects on mitigating changes associated with grain fermentation. In fact, in most cases the combined treatment had little to no effect. This study employed the mixed treatment at up to 10^7^ cells/mL total lactobacilli with each individual species included at an equal concentration. It is possible that utilizing a higher concentration of each individual species could provide different results. Commercial probiotic formulations often contain multiple bacterial species, which are believed to act synergistically to provide health benefits. However, the results of the current study indicate that targeted probiotic therapy may be a better strategy for mitigating grain-induced hindgut acidosis in horses.

It is important to acknowledge that this study was conducted *ex vivo* with a fecal cell suspension model. Previous research has identified small differences (< 1 log) in bacterial enumerations (lactobacilli, GPC, lactate-utilizing bacteria) when comparing fecal material and colonic contents [21]. However, feces are commonly used for *in vitro* digestions, *ex vivo* experiments or bacterial enumeration to approximate microbial changes in the equine hindgut [22, 23, 24, 25, 14, 1, 2]. To our knowledge, no study has been previously conducted to determine the effect of lactobacilli probiotics (most notably *L*. *reuteri*) on microbial changes associated with grain fermentation in horses or any other animal model. Probiotics are defined as live microorganisms that are beneficial to the host [3]. There is evidence for a variety of effects of probiotics on the microbiota and the host. The current results support the hypothesis that lactobacilli exert competitive exclusion among other amylolytic bacteria and antimicrobial allelopathy is implicated. Previous studies report other benefits such as competition for adhesion sites, inhibition of the production of bacterial toxins and improving overall gastrointestinal health [26, 27, 28]. All of the aforementioned benefits and mechanisms of action are mutually compatible. Research is limited and different conclusions are reached regarding the efficacy of the use of probiotics in horses. Future research is needed to evaluate the effect of targeted lactobacilli probiotics on grain fermentation *in vivo*.

## Conclusions

The results from the current *ex vivo* study indicate that exogenous lactobacilli, most notably *L*. *reuteri*, can impact the microbial community composition, fermentation end-products and pH of cereal grain fermentations by equine hindgut microorganisms. These effects were independent of viability; *i*.*e*. autoclaved lactobacilli had the same effects as live. Additionally, the initial rate of starch disappearance with grain fermentation was slower with both live and dead *L*. *reuteri* addition, but these effects were substrate dependent. A slower rate of starch disappearance could permit adaptation of the fecal microflora allowing for greater stability. Thus, fermentations containing added *L*. *reuteri* either had similar or higher total starch disappearance in comparison to substrate only controls. This study provides a potential targeted treatment strategy for grain-induced hindgut acidosis in horses. Future research is needed to evaluate the effect of targeted probiotic therapies *in vivo*.
